# Evaluation de la tenue du partogramme dans une maternité universitaire

**DOI:** 10.11604/pamj.2015.21.99.6047

**Published:** 2015-06-09

**Authors:** Manel Limam, Chekib Zedini, Meriem El Ghardallou, Menel Mellouli, Iheb Bougmiza, Jihène Sahli, Hédi Khairi, Ali Mtiraoui, Thouraya Nabli Ajmi

**Affiliations:** 1 Laboratoire de Recherche « LR12ES03 », Département de Médecine Familiale et Communautaire, Faculté de Médecine Ibn El Jazzar Sousse, Université de Sousse, Tunisie; 2 Service de Gynécologie et Obstétrique, CHU Farhat Hached, Sousse, Tunisie

**Keywords:** Partogramm, évaluation, audit clinique, obstétrique, partograph, evaluation, clinical audit, obstetrics

## Abstract

**Introduction:**

La mortalité maternelle est un problème majeur de santé mondiale. Une grande proportion de ces décès serait évitable par des soins adéquats, une aide à l'accouchement, la disponibilité des soins d'urgence et l'utilisation des outils d'aide à la décision tels que le partogramme. L'objectif était d’évaluer l’écart entre ce qui est censé être fait et ce qui est fait réellement pour les différents paramètres situés dans le partogramme au sein d'une maternité de 3^ème^ niveau et élaborer des recommandations pour la mise en place d'un plan d'action.

**Méthodes:**

Il s'agit d'une étude descriptive rétrospective par audit clinique, effectuée sur un échantillon de 400 dossiers obstétricaux des parturientes ayant accouchées dans la maternité du CHU Farhat Hached durant l'année 2011. Le référentiel utilisé est celui réalisé par l'Agence Nationale d'Accréditation et d'Evaluation en Santé en l'an 2000, concernant la qualité de la tenue du partogramme.

**Résultats:**

La majorité des critères d’évaluation portant sur la présentation du partogramme était conforme. Deux critères concernant la variété de la présentation et le rythme cardiaque fœtal étaient non conformes parmi ceux portant sur la surveillance du fœtus. Plusieurs critères en rapport avec la surveillance de la mère étaient non conformes. Aucun des critères portant sur les traitements administrés et les marqueurs d’évènements n'est conforme. Les critères portant sur la naissance et la surveillance immédiate qui étaient non conformes sont: le début des efforts expulsifs, le mode d'accouchement, l’état du périnée, la délivrance et la révision utérine.

**Conclusion:**

La véritable démarche de l'audit clinique se doit d'aller au-delà du recueil et de l'analyse des données, le but final étant l'amélioration des pratiques.

## Introduction

La mortalité maternelle est l'un des indicateurs accessibles et fiables utilisés par les Nations Unies pour mesurer le développement humain et social. Cet indicateur est aussi le reflet d'une grande disparité entre les pays [1–3]. En 2005, la mortalité maternelle est de 8/100 000 naissances vivantes (NV) dans les pays développés, 450/100 000 NV dans les pays en développement et atteint les 870/100 000 NV dans les pays les moins développés [4, 5]. A partir de ce constat, la mortalité maternelle se présente donc comme un problème majeur de santé mondiale. C'est ainsi que plusieurs initiatives mondiales ont été lancées, dont la plus récente était en l'an 2000, dans le cadre des objectifs du millénaire pour le développement fixés par les Nations Unies [6]. L'amélioration de la santé maternelle était le cinquième objectif et il visait la réduction de trois quarts de la mortalité maternelle entre 2000 et 2015 et la fourniture d'un accès universel aux soins de santé génésique d'ici 2015 [7]. Ce sont les causes obstétricales directes qui dominent largement les étiologies. Il s'avère aussi qu'une grande proportion des décès maternels serait évitable par des soins prénataux et post-nataux adéquats ainsi qu'une aide à l'accouchement et la disponibilité des soins d'urgence pour traiter les complications. De plus l'utilisation des outils d'aide à la décision tels que le partogramme et les protocoles de soins permettent une standardisation des pratiques et une amélioration du pronostic des femmes [2] en améliorant la qualité des soins fournis. Plusieurs maternités ont adopté le partogramme dans leur pratique quotidienne, essentiellement dans les pays en voie de développement [2, 8]. La Tunisie, consciente de l'ampleur et de la gravité de ce problème, dès 1990, a mis en place un programme national de périnatalité, renforcé d'une stratégie de réduction de la mortalité maternelle en 1999 [9] et comme nombreux pays, elle a intégré le partogramme à travers son programme national tout en l'adaptant à ses réalités et son contexte. La Haute Autorité de Santé (HAS), ancienne Agence Nationale d'Accréditation et d'Evaluation en Santé (ANAES) a établi des recommandations en 2000 conjointement à la mise en place d'une évaluation des pratiques professionnelles sur la qualité du partogramme, afin d'identifier les dysfonctionnements encore existants et d'y apporter des axes d'amélioration [10, 11]. C'est dans cette perspective que nous avons mené ce travail pour évaluer les pratiques professionnelles en matière de la tenue du partogramme au niveau de la maternité universitaire du Centre Hospitalier Universitaire (CHU) Farhat Hached de Sousse. Les objectifs de notre travail étant d’évaluer l’écart entre ce qui est censé être fait et ce qui est fait réellement pour les différents paramètres situés dans le partogramme au sein d'une maternité de 3^ème^ niveau en 2011 et d’élaborer des recommandations pour la mise en place d'un plan d'action.

## Méthodes

Il s'agit d'une étude descriptive rétrospective par audit clinique (AC), effectuée sur un échantillon de dossiers obstétricaux des parturientes ayant accouchées dans la maternité du CHU Farhat Hached durant l'année 2011 et qui étaient éligibles au partogramme. Nous avons estimé qu'avoir 400 dossiers à analyser est suffisant. Le total des accouchements en 2011 était de 9957. Une fraction de sondage de 1/25 est utilisée pour tirer au sort les dossiers d'accouchement. Nous avons stratifié notre population d’étude selon le mode d'accouchement. C'est ainsi que nous avons tiré au sort un dossier sur 25 parmi les dossiers d'accouchements par voie basse et parmi les dossiers des césariennes. L’échantillon a porté sur 400 dossiers dont 100 dossiers de césariennes et 300 dossiers d'accouchements par voie basse. Cette répartition a respecté la répartition des accouchements par césarienne et voie basse par rapport au total des accouchements de l'année 2011. Ont été incluses, toutes patientes ayant accouché dans le service durant l'année 2011. Les césariennes programmées ou avant le travail, les accouchements effectués à l'extérieur de la maternité (exemple à domicile) et le suivi de travail et l'accouchement de mort fœtale in utéro n'ont pas été inclus dans l’étude. Le référentiel utilisé est celui réalisé par l'ANAES en l'an 2000, concernant la qualité de la tenue du partogramme (11). Celui-ci est divisé en 6 parties (36 critères) et évalue: la présentation du partogramme (14 critères), le fœtus (5 critères), la mère (14 critères), les traitements (5 critères), les actes et marqueurs d’évènements (16 critères), la naissance et la surveillance immédiate (18 critères). Nous nous sommes aidés de la fiche d'aide à la lecture des critères du guide de l’évaluation de l'ANAES pour le recueil des informations [11]. La conformité de chaque critère du partogramme est analysée par rapport au référentiel de l'ANAES. Un critère conforme entraîne une réponse « oui », un critère non conforme entraîne une réponse « non » et un critère ne pouvant être évalué est qualifié de « non adapté » ou «non applicable» (NA). La saisie et l'analyse des données ont été faites à l'aide du logiciel SPSS.18. Le taux de conformité est représenté par le pourcentage de « oui ». Dans les cas où le critère n'est pas applicable, le taux de conformité est calculé en ramenant la proportion de « oui » aux nombres de partogramme où le taux est applicable c'est à dire 400 moins les « non applicables ». On notera (n)= 400-x(NA). Un critère sera considéré comme correctement appliqué si son taux de conformité est supérieur à 75%.

## Résultats

### Les critères d’évaluation portant sur la présentation du partogramme (Figure 1)

En ce qui concerne la présentation du partogramme, le taux de conformité global était de 91,47%. Seul le critère concernant le nom du médecin pédiatre était inférieur au seuil de 75% (48,5%) et il est ainsi considéré incorrectement appliqué.

**Figure 1:**
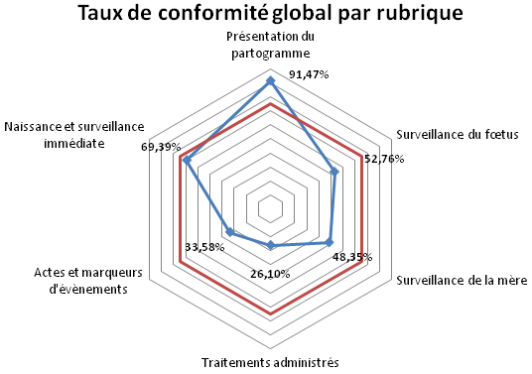
Les taux de conformité globaux par rubrique

### Les critères d’évaluation portant sur la surveillance du fœtus (Figure 1)

Le taux de conformité global des critères de surveillance du fœtus était de 52,76%. Ce faible taux est du à deux critères parmi les quatre qui sont incorrectement appliqués (Tableau 1). Le premier étant la variété de présentation qui n'a été notée que dans 2,75% des cas. Le deuxième concerne le rythme cardiaque fœtal (RCF), qui n'est commenté que dans 9,5% des cas et les commentaires figurent à l'intérieur du dossier obstétrical tandis que dans la feuille du partogramme on se contente de mettre un signe plus (+) dans la case dédiée à l'interprétation du RCF.

**Tableau 1:** Critères d’évaluation portant sur la surveillance du fœtus (4 critères)

Critères	TC (%)
9-La présentation du fœtus est notée	99,5
10-La variété de présentation est notée ou commentée	**2,75**
A la rupture de la poche des eaux	**3,5**
A chaque examen après la rupture	**2,0**
11-Le niveau de la présentation est noté à chaque examen	99,3
12-Le Rythme cardiaque fœtal est commenté à chaque examen	**9,5**

### Les critères d’évaluation portant sur la surveillance de la mère (Figure 1)

Les taux de conformité des critères d’évaluation portant sur la surveillance de la mère variaient entre 0,3% et 99,5% (Tableau 2). Plusieurs critères sont incorrectement appliqués selon ce qui figure dans le référentiel de l'ANAES tels que le comportement de la mère, l’évaluation de sa douleur, l'absence de surveillance de la position du col utérin contrairement aux autres paramètres du col tels que la dilatation, la consistance et la longueur. De même l'estimation de la quantité de liquide amniotique et l'analyse des contractions utérines étaient non conformes.

**Tableau 2:** Critères d’évaluation portant sur la surveillance de la mère (8 critères)

Critères	TC (%)
13-Le comportement de la mère est noté au moins une fois	**3,0**
14-Une évaluation de la douleur est notée à chaque examen	**0,3**
15-La surveillance du col utérin comporte les éléments suivants	**69,8**
Position	**2,0**
Longueur	96,3
Consistance	81,5
Dilatation	99,5
16-L’état de la poche des eaux est noté à chaque examen jusqu’à la rupture	97,1
17-L'aspect du liquide amniotique est noté à l'entrée ou à la rupture	91,6
18-L'estimation de la quantité de liquide amniotique est notée	**24,5**
19-L'analyse des contactions utérines est effectuée à chaque examen	**30,0**
20-Sont notés:	**70,5**
La pression artérielle	99,3
La température	97,0
Le pouls	**15,3**

### Les critères d’évaluation portant sur les traitements administrés (Figure 1)

Les critères d’évaluation portant sur les traitements administrés sont tous au dessous du seuil de bonne application à savoir: l'heure de pose de la voie d'abord (12%), le nom du prescripteur (19,3%), le nom des médicaments (44,8%), la voie d'administration (21,5%) et la posologie (33%).

**Les critères d’évaluation portant sur les actes et les marqueurs (** Figure 1 **)** Les taux de conformité des critères d’évaluation en rapport avec les actes sont très bas pour la majorité des critères (sondage urinaire (2,2%), les péridurales (25%) et l'anesthésie générale (60%). Cependant la rupture des membranes qu'elle soit spontanée ou artificielle a été signalée dans 96,8%. On note aussi un défaut de la signalisation des marqueurs d’évènements dans les dossiers tels que les heures d'appels et d'arrivée des différents intervenants (médecins obstétriciens (1,7%), anesthésiste (2,9%), pédiatre (14%)) ainsi que les heures de décision des césariennes (43,1%) et de transfert au bloc (6,6%).

### Les critères d’évaluation portant sur la naissance et la surveillance immédiate (Figure 1)

Les taux de conformité des critères portant sur la naissance et la surveillance immédiate du post partum sont très disparates avec un taux global de 69,3%. Les données concernant la date, l'heure de naissance et les informations relatives au nouveau-né étaient correctement notées dans le dossier. Tandis que le début des efforts expulsifs était le critère le moins conforme (1,8%). D'autres critères étaient aussi non conformes mais avec des taux assez proches de 75% tels que les informations relatives à la délivrance ses modes et ses indications (Tableau 3).

**Tableau 3:** Critères d’évaluation portant sur la naissance et la surveillance immédiate (9 critères)

Critères	TC (%)
21-Le début des efforts expulsifs	**1,8**
22-Le mode d'accouchement est noté	80,2
Expulsion spontané	**68,0**
Extraction instrumentale et son indication	**61,7**
Césarienne et son indication	93,0
Manœuvres	-
23-L’état du périnée est noté	**70,3**
24-La date et l'heure de naissance sont notées	97,0
25-Le sexe, le poids, l'Apgar et le prénom du nouveau-né sont notés	98,8
26-Le pH du cordon est noté (selon protocole du service)	-
27-L'heure et le mode de délivrance sont notés	**69,5**
34-bis- Si délivrance artificielle-révision utérine, l'indication est notée	**46,0**
28-La révision utérine isolée est notée (selon contexte)	**72,4**
35-bis- Si révision utérine, l'indication est notée	**72,0**
29-La surveillance de l'accouchée est notée et comprend les éléments suivants:	85,9
Globe « de sécurité »	82,7
Pouls, pression artérielle et température	92,5
Volume des pertes sanguines	82,5

## Discussion

D'après l'OMS, la qualité en santé, c'est garantir à chaque patient un ensemble d'actes diagnostiques et thérapeutiques lui assurant le meilleur résultat. Ceci en terme de santé, conformément à l’état actuel de la science médicale, au meilleur coût, pour le même résultat, au moindre risque iatrogénique et pour sa plus grande satisfaction en terme de procédure, de contact humain, à l'intérieur du système de soins. Dans ce contexte et afin d'assurer des soins de qualité et sans risque pour le patient, l’évaluation apparait comme un des meilleurs moyens de répondre à ces besoins. C'est dans ce cadre que la Haute Autorité de Santé en France, s'est chargée de la promotion de bonnes pratiques en développant l'axe de l’évaluation des pratiques professionnelles. Il s'agit certainement d'une démarche indispensable dans la pratique médicale actuelle dont l'enjeu est l'amélioration de la qualité d'autant plus que nombreuses sont les études qui mettent en évidence une disparité des pratiques pas toujours expliquée [12]. Il est évident que la tenue du dossier médical est étroitement liée à la qualité de la prise en charge des patients, c'est-à-dire que la qualité des informations qu'il contient. Une étude faite aux Etats-Unis a montré une corrélation chez les médecins entre la manière de gérer leurs dossiers médicaux et la qualité des soins qu'ils dispensent mais aussi contribue à la rigueur du raisonnement du médecin et améliore la prise de décision [13–15]. Notre étude rentre dans le cadre d'une évaluation des pratiques professionnelles par une approche comparative à un référentiel (audit clinique) pour l’étude de la tenue d'un partogramme qui fait partie du dossier médical de la patiente. En effet ce document peut se définir comme étant: le schéma de la progression du travail; la synthèse des éléments de surveillance maternelle et fœtale durant le travail; un outil d'aide à la décision et à la communication pour les professionnels; un document médico-légal et un support de référence pour l'enseignement, la recherche clinique et l’évaluation des pratiques. La rubrique concernant la présentation du partogramme est d'une grande importance puisque les critères [10] qu'elle contient permettent de bien attribuer le partogramme à une patiente donnée, d'assurer l'identification des professionnels ayant assuré la prise en charge et l'identification horaire de la surveillance du travail qui est d'une importance médico-légale essentielle [10]. Tous les critères d’évaluation étudiés concernant cette rubrique, étaient conformes sauf le critère 5d concernant le nom du médecin. A savoir que dans l'audit clinique ciblé fait par la HAS sur la tenue du partogramme en 2006 [10], un ensemble de critères a été enlevé par rapport au référentiel mis en place en 2000 [11] vu que leur nombre a été jugé trop élevé. Le critère 5d (nom du pédiatre) était parmi ceux qui ne figuraient plus dans la rubrique PARTOten, tout en sachant que les noms dont l'identification était indispensable étaient celui de la sage femme, de l'obstétricien et de l'anesthésiste. Le partogramme étant d'une part un support principal pour la communication entre les différents professionnels de santé impliqués dans l'accouchement et d'autre part un document médico-légal, tous ceux qui peuvent intervenir dans le travail et l'accouchement (la sage-femme, le médecin obstétricien, l'anesthésiste, et au même titre le pédiatre…) sont tenus à s'identifier.

Parmi les quatre critères établis par la HAS pour la surveillance du fœtus, seuls deux étaient conformes dans notre travail: la notation de la présentation du fœtus (critère 9) et la notation du niveau de cette présentation à chaque examen (critère 11). En se comparant aux autres études faites sur l’évaluation de la qualité de tenue du partogramme, on retrouve dans l’étude faite dans le groupe hospitalier Cochin-Vincent-de-Paul à Paris, des taux de conformité se rapportant au critère surveillance du RCF variaient de 67% et 71% si on tient compte du partogramme et du dossier obstétrical mais si on considère seulement le partogramme ce taux ne dépasse pas les 50% [16]; en fait on rapporte l'utilisation d'une terminologie inappropriée avec des adjectifs utilisés tels que: rythme cardiaque fœtal « correct », « bon », « suspect » ou « anormal » sans plus de précisions [16]. De même à Grenoble ce critère était de 62% [17]. Dans une autre étude faite à Nantes en 2007, le critère RCF était conforme à 91% [18] proche de celui de l'audit clinique ciblé de la HAS en 2006 (93%) [10]. Vu que ce critère nécessite une rigueur et précision, nous voyons utile de consacrer une rubrique dans le dossier obstétrical du CHU Farhat Hached de Sousse dans laquelle on adjoint une des classifications de l'interprétation du RCF et ainsi on aura une interprétation standardisée pour toutes les parturientes. Il faut aussi insister à interpréter obligatoirement par écrit sur le dossier même si c'est en surveillance continue et même si c'est normal parce que ça incite à regarder plus attentivement le tracé [19]. En ce qui concerne la variété de la présentation, dans l’étude de Nantes ce critère était non conforme avec des taux aux alentours de 50% [18], tandis qu’à Grenoble ce taux était conforme à 98% [17]. La non-conformité de ce critère dans notre étude et dans d'autres, traduit plutôt une impossibilité et une difficulté de réaliser l'examen en fonction de la dilatation du col et le niveau de la présentation ou aussi un doute plutôt qu'une omission de la part des sages-femmes. Certains auteurs se sont interrogés sur l'intérêt de ce diagnostic pour l'amélioration de la prise en charge [18] et de ce fait et afin d'alléger les critères d’évaluation, la notification de la variété de la présentation du fœtus ne figure plus dans la liste des critères de l'audit clinique ciblé réalisé par la HAS en 2006 [10]. Dans la partie réservée à l’évaluation des critères portant sur la mère, apparait un volet très important qui est l’évaluation du comportement de la mère et l’évaluation de sa douleur à chaque examen. Ces deux critères sont largement non conformes avec des taux respectifs de 3% et 0,3%. A Grenoble, le comportement de la mère était évalué dans 44% des cas et la douleur dans 13% des cas [17]. Dans l’étude faite à Nantes, le comportement n'a été rapporté que dans 5% des cas dans le partogramme manuscrit et 58% dans la version informatisée [18]. Ces informations sont quasiment négligées dans le partogramme et même dans les dossiers obstétricaux. Bien que la prise en charge médicale de la patiente soit importante mais son bien-être l'est tout autant étant donné que son comportement peut parfois nuire à la progression du travail. L'appréciation du comportement de la femme par la sage-femme peut ne pas paraitre telle une priorité mais il nous semble que l'accompagnement fait partie intégrante de la profession de sage-femme et est complémentaire à son rôle obstétrical. Il nous parait ainsi important de sensibiliser les sages-femmes à noter les comportements de la mère pour permettre une prise en charge de la femme dans sa globalité tant sur le plan psychologique que médical. On pourra aussi rajouter dans le partogramme une case dédiée à l’évaluation de la douleur ressentie par la patiente en utilisant la réglette « Evaluation Visuelle Analogique EVA ».

Il parait aussi important de les sensibiliser à un remplissage le plus complet possible lors de la surveillance du col utérin avec toutes ces caractéristiques et ne pas se préoccuper seulement de la dilatation puisqu'il s'agit aussi d’éléments importants dans la progression du travail. De plus pour assurer une surveillance rigoureuse des contractions utérines, nous proposons de détailler plus la case consacrée à cet effet en spécifiant la fréquence et l'intensité; ainsi on accordera plus d'attention à son analyse et on pourra alors déceler plus rapidement les troubles de la cinétique utérine. En ce qui concerne les traitements, aucun des critères n'a été conforme au seuil de 75%. La faible conformité de ces critères ne peut s'expliquer que par le manque de précision de la part des prescripteurs. En effet, les médecins et les sages-femmes devraient signaler obligatoirement leurs noms pour chaque prescription; le nom des médicaments doit être inscrit en clair; certaines abréviations peuvent être acceptées telle que « synto » pour le synthocinon. La voie d'administration doit être explicitée ainsi que la posologie (unité, dilution, débit,‘). Dans le travail fait à Paris sur l’évaluation de la tenue du partogramme, la prescription médicamenteuse était aussi jugée insuffisante et non conforme à la réglementation dans presque la moitié des partogrammes audités [16]. A Grenoble seuls les critères se rapportant aux noms des prescripteurs et aux noms des médicaments étaient conformes à 100% [17]. Il en est ainsi dans l’étude faite à Nantes [18]. Pour résumer, la véritable démarche de l'audit clinique se doit d'aller au-delà du recueil et de l'analyse des données, le but final étant l'amélioration des pratiques et non une énumération des insuffisances de remplissage. D'ailleurs dans son guide sur l'audit clinique ciblé, la HAS insiste sur ce point: la planification, la communication des informations et des résultats aux destinataires ce qui a été fait auprès des sages femmes et des médecins. Par la suite, il y a eu la mise en place d'actions d'amélioration concernaient à la fois la sensibilisation des professionnels de la santé à accorder plus de précision et de rigueur au remplissage du partogramme, de plus quelques modifications dans la présentation du partogramme et du dossier médical sont envisagées.

## Conclusion

Cette évaluation des pratiques professionnelles montre que de nombreuses améliorations restent à apporter dans la tenue du partogramme. Nous avons pu aussi observer qu'il était parfois difficile de réunir tous les éléments composants la surveillance materno-fœtale pendant le travail et le post partum immédiat sur le partogramme en tenant compte des recommandations extrêmement nombreuses. D'une part il était question d'un manque de précision et de rigueur de la part des différents membres de l’équipe dans le remplissage de certains critères par négligence, méconnaissance ou oubli. D'autre part, certains critères étaient difficiles à appliquer ou manquaient d'une véritable pertinence clinique. Ainsi cette évaluation ne peut se restreindre à l’étude de ces taux de conformité mais il parait nécessaire de prendre en compte le contexte, les objectifs, les moyens et les acteurs dans leur globalité.
